# Studies on the fitness characteristics of *w*Mel- and *w*AlbB-introgressed *Aedes aegypti* (Pud) lines in comparison with *w*Mel- and *w*AlbB-transinfected *Aedes aegypti* (Aus) and wild-type *Aedes aegypti* (Pud) lines

**DOI:** 10.3389/fmicb.2022.947857

**Published:** 2022-08-05

**Authors:** Candasamy Sadanandane, Kasinathan Gunasekaran, Devaraju Panneer, Sarala K. Subbarao, Manju Rahi, Balakrishnan Vijayakumar, Velan Athithan, Annamalai Sakthivel, Sundaram Dinesh, Purushothaman Jambulingam

**Affiliations:** ^1^ICMR-Vector Control Research Centre, Medical Complex, Puducherry, India; ^2^Indian Council of Medical Research, Ramalingaswami Bhawan, New Delhi, India

**Keywords:** *Wolbachia*, *w*Mel, *w*AlbB, fitness cost, dengue, *Aedes aegypti*

## Abstract

*Wolbachia*, an intracellular maternally transmitted endosymbiont, has been shown to interfere with the replication of dengue virus in *Aedes aegypti* mosquitoes. The *Wolbachia*-transinfected *Ae. aegypti* has been currently released in many countries to test its effectiveness in preventing the transmission of dengue virus. ICMR-Vector Control Research Centre in collaboration with World Mosquito Program Monash University, Australia, has generated two new *Wolbachia*-introgressed *Ae. aegypti* Puducherry (Pud) lines *via* backcrossing *Ae. aegypti* females of Australian (Aus) strains, infected with *w*Mel and *w*AlbB *Wolbachia* with wild-type *Ae. aegypti* Puducherry (Pud) males. *Wolbachia* infections are known to induce a fitness cost and confer benefit on the host mosquito populations that will influence spread of the *Wolbachia* into native wild mosquito populations during the field release. Hence, the induced fitness cost or benefit/advantage in the two newly generated *Ae. aegypti* (Pud) lines was assessed in the laboratory in comparison with the wild-type *Ae. aegypti* (Pud) strain. In addition, maternal transmission (MT) efficiency, induced cytoplasmic incompatibility (CI), and insecticide resistance status of the two (Pud) lines were determined to assess the likely frequency of *w*Mel and *w*AlbB infections in the native wild population after field invasion. The study shows that *w*Mel and *w*AlbB infections did not induce any fitness cost on the two newly generated (Pud) lines. Rather, in terms of wing length, fecundity, egg hatch rate, and adult survival, the *Wolbachia* introgression conferred fitness benefits on the (Pud) lines compared to uninfected *Wolbachia* free wild *Ae. aegypti* population. *w*Mel and *w*AlbB exhibited a high maternal transmission (99–100%) and induced nearly complete (98–100%) cytoplasmic incompatibility. Both the (Pud) lines were resistant to deltamethrin, malathion, DDT, and temephos, and the level of resistance was almost the same between the two lines as in the wild type. Overall, the stable association of *w*Mel and *w*AlbB established with *Ae. aegypti* and the reproductive advantages of the (Pud) lines encourage a pilot release in the field for population replacement potential.

## Introduction

Dengue is the fastest spreading arboviral infection of humans accounting for a considerable disease burden across the tropics. It is estimated that approximately 390 million dengue infections and 96 million cases occur worldwide annually (World Health Organization [WHO], 2022). In India, it is endemic in 28 States and six union territories and is the leading cause of febrile illness (Ganeshkumar et al., 2018). A total of 913,817 cases of dengue and 1,490 deaths were reported in the country during 2015–2021 (National Vector Borne Disease Control Programme [NVBDCP], 2022). Dengue virus (DENV) is transmitted by infective bite primarily of *Aedes aegypti* females and secondarily of *Aedes albopictus*. No specific drugs are currently available to treat the dengue patients. The safety concerns with the currently available vaccines prevented their use for prophylaxis in the public health program (Wilder-Smith et al., 2010; Deng et al., 2020). Control of the vector mosquito is thus the only option to prevent and interrupt the transmission of dengue virus. Use of chemical larvicides, biological control agents, thermal fogging, and source reduction activities are the ongoing practical measures of vector control today, but these measures have yielded only a limited success in reducing dengue cases not only in India (Jain and Sharma, 2017), but also in other countries (Kay and Vu, 2005; Garcia et al., 2019). In addition, these interventions require repeated applications and are expensive and difficult to carry out in urban areas (Iturbe-Ormaetxe et al., 2011). This has necessitated the testing of alternative methods to achieve effective control of dengue in India.

One such method is the use of *Wolbachia*-based strategy to prevent the transmission of dengue and other arboviral infections. *Wolbachia* is an endosymbiotic intracellular gram-negative bacterium that is naturally prevalent in about 51% (CI: 48–57) of insect species (Weinert et al., 2015) and 39.5% of the 147 mosquito species screened (Bourtzis et al., 2014). *Wolbachia pipientis* (*w*Pip) is the strain first isolated from the mosquito species *Culex pipiens* (Hertig and Wolbach, 1924). Other mosquito species known to be naturally infected with *Wolbachia* are *Culex quinquefasciatus* (Dumas et al., 2013), *Aedes fluviatilis* (Moreira et al., 2009), and *Ae. albopictus* (Sinkins et al., 1995). Recent studies have reported the presence of *Wolbachia* in *Ae. aegypti* with a low frequency and density (Teo et al., 2017; Bennett et al., 2019; Carvajal et al., 2019; Kulkarni et al., 2019), but the evidence is not compelling (Ross et al., 2020).

*Wolbachia* alters reproductive fitness of arthropod vectors through selective male killing, parthenogenesis, feminization of male embryos, and cytoplasmic incompatibility (CI) (Werren et al., 2008). Of these, CI is the prominent and is the only phenotype found in mosquitoes (Sinkins, 2004). It also alters the vector competence of transinfected arthropod vectors for the transmission of arboviruses through competition for resources, such as cholesterol (Hedges et al., 2008; Glaser and Meola, 2010), pre-activation of the immune system (immune-priming), induction of the phenol-oxidase cascade, and stimulation of microRNA-dependent immune pathways, that are essential for host defense against viruses (Sim et al., 2014; Johnson, 2015).

Some *Wolbachia* strains have many traits that induce fitness costs on their host mosquito populations (Allman et al., 2020) and that will affect its spread among the native wild population during field release. The most pathogenic *Wolbachia* strain *w*MelPop generates large deleterious effects on adult longevity, egg viability, and reproductive potential (McMeniman and O’Neill, 2010; Almeida et al., 2011; Caragata et al., 2016). However, these deleterious effects are less pronounced or absent with *w*Mel and *w*AlbB strains of *Wolbachia* (Axford et al., 2016).

Transinfection of *Ae. aegypti* with *Wolbachia* strains, *w*MelPop, *w*Mel, and *w*AlbB, has initially been shown to significantly reduce its vector competence, particularly to dengue virus under laboratory conditions (Moreira et al., 2009; Frentiu et al., 2010; Ant et al., 2018). In small-scale field releases of transinfected *Ae. aegypti*, *Wolbachia* was found to spread in high frequencies among the native *Ae. aegypti* populations (O’Neill et al., 2018; Indriani et al., 2020; Ryan et al., 2020) and suppress replication of dengue virus in mosquitoes (Ferguson et al., 2015). *Wolbachia*-infected *Ae. aegypti* mosquitoes can be deployed for either population replacement or population suppression. Both approaches rely on CI induced by *Wolbachia*. The population replacement approach involves the release of both male and female *Wolbachia*-infected mosquitoes that reduce virus transmission in *Ae. aegypti* populations. Population suppression approach involves the release of only males that cannot produce viable offspring when they mate with wild females.

Field trials have been carried out releasing *Wolbachia*-infected males in Singapore (National Environmental Agency [NEA], 2022), California (Crawford et al., 2020), Australia (Beebe et al., 2021), and Puerto Rico (Martín-Park et al., 2022) for population suppression. Currently, the field release of *w*Mel-infected *Ae. aegypti* for population replacement is now underway in 11 countries to evaluate its effectiveness in controlling dengue (World Mosquito Program [WMP], 2022). This strain has successfully been established in Australia (O’Neill et al., 2018; Ryan et al., 2020), Brazil (Pinto et al., 2021), Indonesia (Utarini et al., 2021), and Vietnam (Hien et al., 2022), while *w*AlbB was successfully established among wild mosquito population in Malaysia for population replacement (Nazni et al., 2019). In a recent randomized control trial in Yogyakarta city, Indonesia, field releases of *w*Mel-infected *Ae. aegypti* mosquitoes significantly reduced the virologically confirmed dengue cases by 77.1% in the intervention clusters compared to the control clusters (Utarini et al., 2021). In other city-wide field trials, 76% reduction in dengue cases was observed in Indonesia (Indriani et al., 2020), more than 70% reduction in Brazil (Pinto et al., 2021), 86% reduction in Vietnam (Burki, 2020), and 96% reduction in Australia (O’Neill et al., 2018; Ryan et al., 2020).

Indian Council of Medical Research-Vector Control Research Centre (ICMR-VCRC), Puducherry, India, in collaboration with the World Mosquito Program (WMP) [formerly known as Eliminate Dengue Program (EDP)] from Monash University, Australia, has generated two new *Wolbachia*-introgressed *Ae. aegypti* Puducherry (Pud) lines that carry *w*Mel and *w*AlbB *Wolbachia* strains *via* backcrossing *w*Mel and *w*AlbB *Ae. aegypti* Australia (Aus) females with *Ae. aegypti* Puducherry (Pud) wild males over six generations. The two Indian *Ae. aegypti* (Pud) lines infected with *w*Mel or *w*AlbB *Wolbachia* strains were developed with an aim of testing them, in future, in the field to select the suitable strain for population replacement for Indian conditions.

Before launching a field release of *Wolbachia-*introgressed *Ae. aegypti*, the critical issue that needs to be considered is the fitness of mosquitoes since these mosquitoes must compete effectively with the *Ae. aegypti* wild population to facilitate the efficient invasion of *Wolbachia* into the wild mosquito population through near-complete maternal transmission (MT) coupled with strong CI, but without producing any fitness cost (Fraser et al., 2017). Apart from these factors, male mating competitiveness and vector competence are other important factors which affect the successful establishment of the inherited *Wolbachia* infections in the wild population. In this study, the fitness of the two newly generated *Ae. aegypti* (Pud) lines infected with *w*Mel and *w*AlbB infections was assessed in comparison with that of the wild-type *Ae. aegypti* (Pud) line in terms of life-history traits, such as wing length, fecundity, egg hatch rate, and adult survival. In addition, MT efficiency, induced CI, and insecticide resistance status of the (Pud) lines were determined.

## Materials and methods

### Mosquito strains and colony maintenance

#### Mosquito strains

The eggs of the two *Ae. aegypti* Australian (Aus) lines, one infected by embryonic microinjection with *w*Mel *Wolbachia* isolated from *Drosophila melanogaster* (Walker et al., 2011) and the other infected by microinjection with *w*AlbB *Wolbachia* infection from *Ae. albopictus* (Xi et al., 2005) imported from the World Mosquito Program (WMP), Australia, were used to raise colonies of the two (Pud) lines. Eggs of wild-type *Ae. aegypti* (Pud) line were collected using ovitraps from different sites in Puducherry, reared to adults, fed with human blood, and allowed for oviposition. The F1 eggs were reared to adults, which were identified and confirmed to be *Ae. aegypti* (Barraud, 1934). The F1 generation adults were used for backcrossing with the *Wolbachia*-infected *Ae. aegypti* (Aus) lines and other laboratory studies on fitness characteristics, MT, CI, sensitivity to heat stress, male mating competitiveness, and population replacement. Prior to the experiments, the wild *Ae. aegypti* (Pud) were screened by PCR assays to ensure that they are free from natural *Wolbachia* infection (Noda et al., 1997).

#### Generation of *Wolbachia*-infected Indian strains

Backcrossing was done in three replicates. Into each replicate cage containing 250 *w*Mel or *w*AlbB *Ae. aegypti* (Aus) females, 250 wild-type *Ae. aegypti* (Pud) males were released for mating [Backcross I (BC 1)]. Five days after mating, the females were fed with human blood and allowed to oviposit, and the eggs were collected and stored. One-week-old eggs obtained from backcross I (BC I) were hatched replicate wise and reared to adults. The adult female progeny from BC I were backcrossed with wild-type F1 males (BC II). In total, for each *Wolbachia* strain, six backcrossing were done. After the sixth backcrossing, the resultant *Ae. aegypti* colonies were designated as the *w*Mel *Ae. aegypti* (Pud) and *w*AlbB *Ae. aegypti* (Pud) lines. At every generation, females of the (Pud) lines were outcrossed (250 females and 225 males of respective line) with 10% (25) wild-type (Pud) males to minimize the selection pressure due to continuous rearing under laboratory conditions over many generations.

#### Colony maintenance

*w*Mel- and *w*AlbB-infected *Ae. aegypti* (Aus) lines, *w* Mel-, and *w*AlbB-infected *Ae. aegypti* (Pud) lines and uninfected *Ae. aegypti* (Pud) line were reared in the insectary at 27 ± 2°C temperature and 80 ± 10% relative humidity with a photoperiod of 12L and 12D. Colonies of 500 adults (1:1 sex ratio) were maintained in BugDorm cages (W30 x D30 x H30 cm) (BugDorm Stores, Australia). To provide nutrition and hydration, the adult mosquitoes were provided with 10% sucrose solution kept in sugar cups. Five-day-old females were fed with human blood obtained from blood bank (Pondicherry AIDS Control Society, Government of Puducherry, India) through artificial membrane feeding system following the SOP on blood feeding (World Mosquito Program [WMP], 2018a). Three days after blood feeding, oviposition cups (plastic polycarbonate cups, 200 ml capacity) lined inside with filter paper and half-filled tap water were placed in the adult mosquito cages for 2 days. On the third day, the egg papers were removed from the oviposition cups and allowed to dry at room temperature (27°C ± 2°C; 80 ± 10% RH) for 2 days. Once the egg papers were dried, they were transferred to plastic ziplock bags and stored in a sealed container at 27°C ± 2°C and 80% RH using saturated KCL solution (Ross et al., 2017; World Mosquito Program [WMP], 2017). The tap water supplied by the municipality was used for both egg hatching and larval rearing. The quality of the tap water was checked periodically (every 6 months) by subjecting it to physical, chemical, and bacteriological analysis (Water Testing Laboratory, Level II + Category, Government of Puducherry). Synchronous hatching of eggs was done using cooled boiled (deoxygenated) water containing brewer’s yeast (0.2 g/l) in an airtight container (Judson, 1960; Imam et al., 2014; World Mosquito Program [WMP], 2017). One day after hatching, approximately 150 first instar larvae were transferred to enamel trays (45 cm L × 30 cm W × 5 cm H) containing 3 L of tap water (one larva/20 ml). The larvae were fed with fish food (TetraMin Tropical Tablets) at the rate of 2.0 mg/larva. Five days later, pupae were collected; male and female pupae were separated manually and transferred to separate containers (500 ml capacity) that were kept inside one-cubic foot BugDorm cages for adult emergence.

#### Wing length

Mosquito wing size is used to estimate adult body size (Joshi et al., 2014), and body size is considered as an indicator of fitness characteristics of mosquitoes (Xue et al., 2010). Accordingly, the wing size was measured as a part of assessing the fitness of the colonized *w*Mel *Ae. aegypti* (Pud) and *w*AlbB *Ae. aegypti* (Pud) lines. One-day-old 25 unfed males and 25 unfed females of each of the *Ae. aegypti* (Pud) lines from first outcross generation (OCG1) and wild-type *Ae. aegypti* (Pud) line (F1) were killed by freezing at −20°C for 10 min. Subsequently, the right wing of each mosquito was removed, placed on a microslide, and covered with a cover glass. Wings were freed from scales by carefully sliding the cover glass over the microslide. The length from the axillary incision (Alular notch) to the wing tip was measured using a stereomicroscope (Olympus SZ61) attached with a digital camera (Olympus DP22) and measurement software (Cell Sens Entry 1.13).

#### Fecundity and egg hatch rate

Fecundity and egg hatch rate are the two major reproductive biological characteristics, often used to assess the fitness of colonized mosquito populations. Fifty *w*Mel *Ae. aegypti* (Pud) females (OCG1) were crossed with 50 *w*Mel *Ae. aegypti* (Pud) males (OCG1) in six replicates. Similarly, replicate cages were set up with *w*AlbB *Ae. aegypti* (Pud) (OCG1) line and also *w*Mel *Ae. aegypti* (Aus) (F12) and *w*AlbB *Ae. aegypti* (Aus) (F12) lines. For comparison, 50 *Ae. aegypti* (Pud) wild females (F1) were crossed with 50 wild males (F1) in six replicates. In each replicate, 5-day-old females were fed and the blood-fed mosquitoes were allowed to oviposit. The mortality of females was scored daily until oviposition. The number of eggs laid in each replicate was counted using stereomicroscope, and from this count, the average number of eggs laid by a single female was estimated. Eggs obtained from each replicate (*n* = 1,000) were hatched (vide Colony maintenance), and the number of first instar larvae hatched in each replicate was counted to determine the hatch rate.

#### Adult survival

Adult survival of *w*Mel and *w*AlbB *Ae. aegypti* (Pud) lines (OCG9), the *w*Mel, and *w*AlbB *Ae. aegypti* (Aus) parental lines (F19 generation), and the wild-type *Ae. aegypti* (Pud) line (F1 generation) was estimated, simultaneously using batches of 100 adult mosquitoes (1:1 sex ratio), replicated six times. One-week-old eggs of these lines were hatched, and the larvae were reared to pupae (vide Colony maintenance). Male and female pupae were separated. In each replicate, 50 male and 50 female pupae were kept in enamel bowls (300-ml capacity) containing 200 mL of tap water and placed in BugDorm cages (W30 × D30 × H30 cm) for emergence. Adults were provided with 10% sucrose solution. Females were fed weekly on human blood for the entire duration of the experiment. Each time, the same batch of human blood was used for feeding all lines and in all replicates. Three days after each blood feeding, a 300-ml cup containing 150 ml of tap water lined with a filter paper was kept inside the cage for oviposition. The adults were maintained in the insectary at 27 ± 2°C temperature and 80 ± 10% relative humidity. Mortality was scored for males and females daily until all adult mosquitoes had died.

#### Maternal transmission

*Wolbachia* is transferred maternally from an infected female mosquito to her progeny. Maternal transmission is one of the key parameters that influence *Wolbachia* functions in the population replacement process (Hoffmann et al., 1990). Fifty each of *w*Mel and *w*AlbB *Ae. aegypti* (Pud) females were allowed separately to mate with 50 *Ae. aegypti* (Pud) wild males in three replicates to confirm the maternal transmission of *Wolbachia* to their progeny. For comparison, the *w*Mel and *w*AlbB *Ae. aegypti* (Aus) females were allowed separately to mate with the wild-type *Ae. aegypti* (Pud) males. In each replicate, 5-day-old females were fed with the same source of human blood and allowed to oviposit, and the parental adults were screened for *Wolbachia* frequency by real time*-*PCR assays. In each replicate, eggs (progeny) were reared to adults and 160 females from each replicate were screened for the presence of *Wolbachia*. DNA from individual mosquito was extracted by homogenizing in squash buffer. The homogenate was briefly centrifuged, and the supernatant containing DNA was used for the *Wolbachia* diagnosis following the Diagnostics SOP (World Mosquito Program [WMP], 2018b). *w*Mel was screened using primers and probes specifically targeting the *Wolbachia* surface protein (*WSP)* gene (WspTM2_FW: 5′-CATTGGTGTTGGTGTTGGTG-3′; WspTM2_RV: 5′-ACA CCAGCTTTTACTTGACCAG-3′; WspTM_Probe: 5′- LC640- TCCTTTGGAACCCGCTGTGA ATGA-lowaBlack-3′), and *w*AlbB was identified using primers and probes directed to *w*AlbB specific Ankyrin repeat domain gene (*w*AlbB_16009_ FW: 5′-AGTAGTGCAGCGAGTCT-3′; *w*AlbB 16009_RV: 5′-TGGAGGAAGAGTTCACTGTGC-3′; *w*AlbB 16009_Probe: 5′-FAM-ZEN-AATTATCCCCTACCA AAGCAATTAAGATAGAAT-IowaBlack-3′). Gene encoding ribosomal protein (*RPS17*) of *Ae. aegypti* was used as internal positive control (Rps17_FW: 5′-TCCGTGGTATC TCCATCAAGCT-3′; Rps17_RV: 5′-CACTTCCGGCACGTA GTTGTC-3′; Rps17_TaqM_Probe: 5′-HEX-CAGGAGGAGGA ACGTGAGCGCAG-BHQ1-3′). The frequency of *Wolbachia* was calculated as the percentage of positives among the total number of mosquitoes tested. The experiment was repeated three times with successive outcrossed generations (OCG1, OCG2, and OCG3).

#### Cytoplasmic incompatibility

*Wolbachia*-induced cytoplasmic incompatibility is the most commonly occurring reproductive manipulation phenotype that leads to the production of sterile offspring. The crosses between uninfected wild (Pud) females and *w*Mel-infected *Ae. aegypti* (Pud) males were set up in three replicates. Similarly, the wild-type (Pud) females were allowed to mate with the *w*AlbB-infected *Ae. aegypti* (Pud) males. The experiments were repeated three times with successive outcrossed generations (OCG1, OCG2, and OCG3). The eggs collected from the CI crosses were floated for hatching, and the hatch rates were estimated to determine the cytoplasmic incompatibility.

#### Insecticide resistance

In areas reported with dengue infection, insecticides are used to reduce the vector (*Ae. aegypti*) population. For considering the field release of *Wolbachia*-infected *Ae. aegypti* (Pud) lines, it is essential that the (Pud) lines match with the wild population of *Ae. aegypti* in terms of insecticide susceptibility/resistance status. Therefore, the insecticide susceptibility/resistance status of newly generated *w*Mel and *w*AlbB *Ae. aegypti* (Pud) lines was determined through WHO tube assays using impregnated papers at the discriminating concentration of deltamethrin 0.03%, malathion 0.08%, and DDT 4%, the insecticides commonly used in the public health program, in comparison with wild *Ae. aegypti* (Pud) line. The tube assays were done as per the WHO guidelines (World Health Organization [WHO], 2016). The assays were replicated three times with different batches of mosquitoes. Furthermore, intensity assays were carried out at 5X concentrations (5 times higher than the diagnostic concentration) of deltamethrin (0.15%) and malathion (4%) (World Health Organization [WHO], 2016). Susceptibility/resistance status of the larvae of *Ae*. *aegypti* (Pud) lines and of the wild *Ae. aegypti* to temephos, an organophosphorus larvicide used in the control program, was also determined at the diagnostic concentration (0.02 ppm) following the WHO guidelines (World Health Organization [WHO], 2005).

#### Data analysis

Data on wing length, fecundity, and egg hatch (fertility) rates were expressed as mean ± SE. The difference in the wing length between the lines was analyzed using one-way ANOVA as the data on wing length followed normal distribution, and the Bonferroni test was used for pair-wise comparison. The data on fecundity were analyzed using negative binomial regression (variance greater than mean), and the fertility was analyzed using Poisson regression (variance equal to mean) to find out the differences between the strains. Kaplan–Meier survival curve plots were made separately for males and females of each line. The mean duration of 50% adult survival between the lines was analyzed using the log-rank test. Cox-proportional hazard regression model was used to find out the death-risk rate between the lines separately for males and females, and the hazard ratio (HR) with 95% confidence interval was reported. The dose–response data from insecticide susceptibility bioassays were analyzed using probit regression analysis to determine LC_50_ and LC_99_._9_ values. *P*-value < 0.05 was considered as statistically significant. The statistical software STATA 14.2 (Texas, United States) and SPSS 16.0 were used for data analysis.

## Results

### Wing length

The mean wing length of *w*Mel *Ae. aegypti* (Pud) females (*n* = 25) was 3.03 ± 0.02 mm and that of the males (*n* = 25) was 2.34 ± 0.01 mm. The mean wing length of *w*AlbB (Pud) females and males was 3.07 ± 0.01 mm and 2.35 ± 0.01 mm, respectively, and those of wild (Pud) females and males were 2.96 ± 0.02 mm and 2.30 ± 0.01 mm, respectively. Analysis of variance showed that the wing length of males [*F* = 5.65, *df* (2.72), *p* = 0.005] and females [*F* = 14.26, *df* (2.72), *p* < 0.001] varied significantly between the lines. Pair-wise comparisons showed that the wing length of females of both the introgressed *Ae. aegypti* (Pud) lines was significantly greater than that of wild *Ae. aegypti* (Pud) females (*w*Mel (Pud) female: *p* = 0.004; *w*AlbB (Pud) female: *p* < 0.001). In case of males, *w*AlbB (Pud) line showed greater wing length than that of wild *Ae. aegypti* (Pud) (*p* = 0.005) line. Wing length in males (*p* = 0.836) and in females (*p* = 0.176) did not differ significantly between the two *Ae. aegypti* (Pud) lines.

### Fecundity and egg hatch rate

**FIGURE 1:**
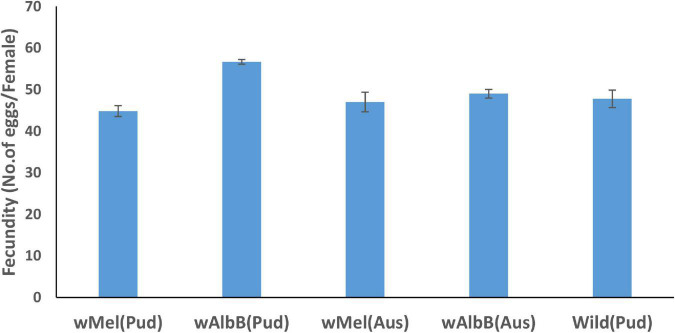
Fecundity (average number of eggs per female ± SE) of *Wolbachia*-infected *Ae. aegypti* females and uninfected wild *Ae. aegypti* (Pud) females.

The fecundity (average number of eggs laid by a female) of *w*AlbB *Ae. aegypti* (Pud) line was significantly [Incidence rate (IR) = 1.23, *p* < 0.001] greater than those of the wild-type *Ae. aegypti* (Pud) and *w*Mel *Ae. aegypti* (Pud) lines (Figure 1). When compared to wild *Ae. aegypti* (Pud), the fecundity of *w*Mel *Ae. aegypti* (Pud), *w*Mel *Ae. aegypti* (Aus), and *w*AlbB *Ae. aegypti* (Aus) did not differ significantly (*p* > 0.05) (Figure 1).

**FIGURE 2:**
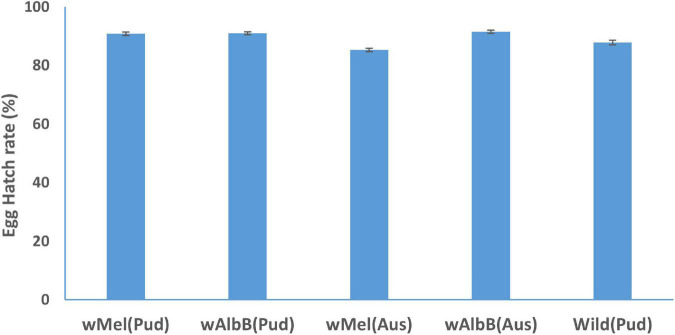
Hatch rate (± SE) of eggs of *Wolbachia*-infected *Ae. aegypti* females and uninfected wild *Ae. aegypti* (Pud) females.

The egg hatch rate of *w*AlbB (Aus) line was significantly (IR = 1.04, *p* = 0.035) greater than that of wild-type (Pud) line (Figure 2). The egg hatch rates of *w*Mel (Pud) (IR = 1.03, *p* = 0.08), *w*AlbB (Pud) (IR = 1.03, *p* = 0.068), and *w*Mel (Aus) (IR = 0.97, *p* = 0.136) lines did not differ significantly when compared to the wild-type (Pud) line (Figure 2).

### Adult survival

**FIGURE 3:**
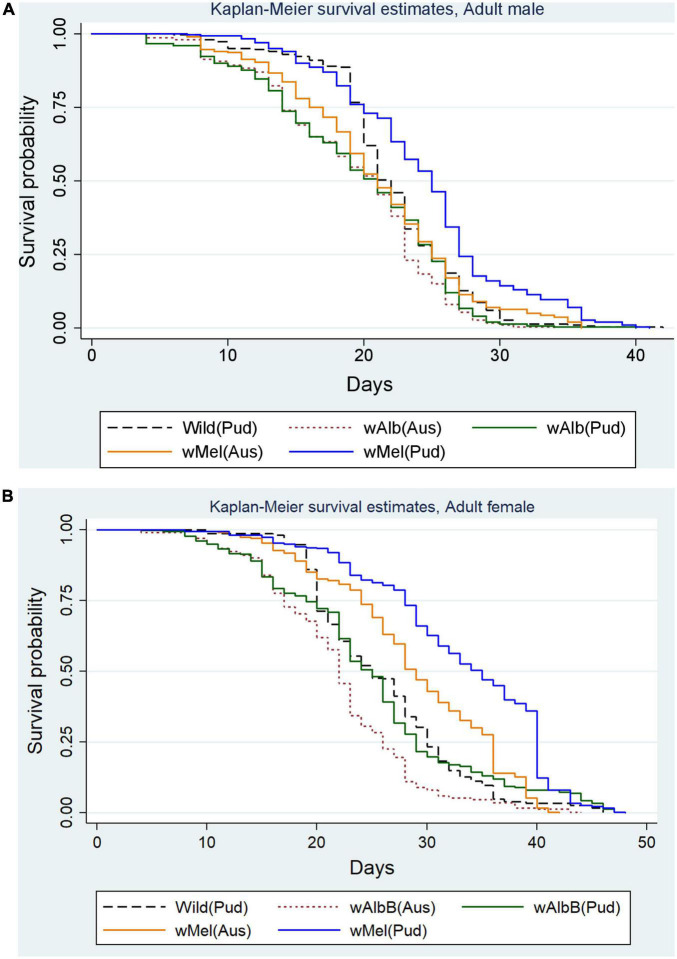
**(A)** Survival of *Wolbachia-*infected *Ae. aegypti* males in comparison with uninfected wild *Ae. aegypti* (Pud) males. **(B)** Survival of *Wolbachia*-infected *Ae. aegypti* females in comparison with uninfected wild *Ae. aegypti* (Pud) females.

The effects of *w*Mel and *w*AlbB infections on the survival of *Ae. aegypti* (Pud) lines over time were examined. In *w*Mel *Ae. aegypti* (Pud) line, 50% of males survived up to 25 days and females up to 35 days (Figures 3A,B), whereas in *w*Mel (Aus) line, 50% of males and 50% of females survived up to 21 and 29 days, respectively (Figures 3A,B).

In *w*AlbB (Pud) line, 50% of adult males survived up to 21 days and females up to 25 days and the corresponding values for *w*AlbB (Aus) line were 21 days for males and 22 days for females. In the case of wild (Pud) line, survival of 50% males was up to 22 days and females up to 25 days (Figures 3A,B).

The mean duration of 50% survival of the *w*Mel *Ae. aegypti* (Pud) males and females was significantly greater than those of the other *Wolbachia*-infected and the uninfected wild *Ae. aegypti* males and females (*p* < 0.001; using log-rank test). Overall, the survival curves of males (*p* < 0.001, log-rank test) and females (*p* < 0.001, log-rank test) of the five lines varied significantly. Among the adult *Ae. aegypti* males, the risk of death was significantly lower in the *w*Mel *Ae. aegypti* (Pud) males (HR = 0.66, 95% CI: 0.56–0.78, *p* < 0.001) than that of the wild *Ae. aegypti* (Pud) males. The risk of death was significantly higher in the *w*AlbB *Ae. aegypti* (Pud) line (HR = 1.25, 95% CI: 1.06–1.47, *p* = 0.007) and the *w*AlbB *Ae. aegypti* (Aus) line (HR = 1.43, 95% CI: 1.22–1.68, *p* < 0.001), and the risk of death was higher but not significant in the *w*Mel *Ae. aegypti* (Aus) line (HR = 1.06, 95% CI: 0.90–1.24, *p* = 0.505) when compared to that of the wild *Ae. aegypti* (Pud) line. Among the adult females, the risk of death was significantly lower in the *w*Mel *Ae. aegypti* (Pud) females (HR = 0.44, 95% CI: 0.38–0.52, *p* < 0.001) and the *w*Mel *Ae. aegypti* (Aus) females (HR = 0.73, 95% CI: 0.62–0.86, *p* < 0.001) than that of the wild *Ae. aegypti* (Pud) females. The risk of death was lower, but not significantly different in *w*AlbB *Ae. aegypti* (Pud) line (HR = 0.92, 95% CI: 0.78–1.08, *p* = 0.301) than that of the wild *Ae. aegypti* (Pud) line. The risk of death was significantly higher in the *w*AlbB *Ae. aegypti* (Aus) line (HR = 1.65, 95% CI: 1.40–1.94, *p* < 0.001) than that of the wild *Ae. aegypti* (Pud) line.

### Maternal transmission

The maternal transmission efficiency of the *w*Mel and *w*AlbB (Pud) lines was studied in comparison with that of the *Wolbachia*-infected (Aus) parental lines by testing the frequency of infected adult progeny produced by an infected female. In all the replicates of *w*Mel and *w*AlbB *Ae. aegypti* (Pud) lines, a near-complete maternal transmission was observed with a frequency of *Wolbachia* in the adult progeny ranging from 99 to 100% (Table 1), while for *w*Mel and *w*AlbB *Ae. aegypti* (Aus) lines, the frequency of *Wolbachia* in the adult progeny ranged from 96 to 100% (Table 2).

**TABLE 1:** Maternal transmission of *Wolbachia* assessed from the progeny of crosses between *w*Mel-infected *Ae. aegypti* females and uninfected wild *Ae. aegypti* males.

Crosses	Generation	*Wolbachia* frequency in progeny (%)^#^		
		Replicate 1 (%)	Replicate 2 (%)	Replicate 3 (%)
*w*Mel *Ae. aegypti* (Pud) ♀× wild *Ae. aegypti* (Pud) ♂	OCG*1	99.4	100	100
	OCG 2	100	100	100
	OCG 3	100	100	100
*w*Mel *Ae. aegypti* (Aus) ♀ **×** wild *Ae. aegypti* (Pud) ♂	OCG 1	100	100	100
	OCG 2	98.0	100	98.0
	OCG 3	98.0	99.0	98.0

^#^At every generation, 160 adult female progeny from each replicate were screened for Wolbachia.

*OCG, outcross generation.

**TABLE 2:** Maternal transmission of *Wolbachia* assessed from the progeny of crosses between *w*AlbB-infected *Ae. aegypti* females and uninfected wild *Ae. aegypti* males.

Crosses	Generation	*Wolbachia* frequency in progeny (%)^#^		
		Replicate 1 (%)	Replicate 2 (%)	Replicate 3 (%)
*w*AlbB *Ae. aegypti* (Pud) ♀ **×** wild *Ae. aegypti* (Pud) ♂	OCG*1	100	99.0	99.4
	OCG 2	100	100	100
	OCG 3	100	99.0	99.4
*w*AlbB *Ae. aegypti* (Aus) ♀ **×** wild *Ae. aegypti* (Pud) ♂	OCG 1	100	100	97.0
	OCG 2	100	96.3	100
	OCG 3	100	99.1	100

^#^At every generation, 160 adult female progeny from each replicate were screened for Wolbachia.

*OCG, outcross generation.

### Cytoplasmic incompatibility

Cytoplasmic incompatibility was determined by measuring the egg hatch rate from crosses between the uninfected wild *Ae. aegypti* (Pud) females and the *Wolbachia*-infected males. Egg hatch rate of the crosses between uninfected wild *Ae. aegypti* (Pud) females and *w*Mel-infected *Ae. aegypti* (Pud) males was only 0.2–1.2% in the first outcross generation (OCG 1). In outcross generations 2 and 3, no viable offspring were obtained indicating a complete sterility (Table 3). Similarly, the crosses between the uninfected wild *Ae. aegypti* (Pud) females and the *w*AlbB-infected *Ae. aegypti* (Pud) males resulted in complete sterility as there was no viable offspring in all the three generations tested (Table 4).

**TABLE 3:** Induced cytoplasmic incompatibility observed in crosses between uninfected wild *Ae. aegypti* (Pud) females and *w*Mel-infected *Ae. aegypti* (Pud) males.

Generation	Replicate^#^	No. of eggs laid	No. hatched	Egg hatch rate (%)
OCG*1	1	1,637	3	0.2%
	2	1,096	13	1.2%
	3	1,101	8	0.7%
OCG 2	1	632	0	0%
	2	872	0	0%
	3	1,157	0	0%
OCG 3	1	543	0	0%
	2	608	0	0%
	3	851	0	0%

^#^In each replicate, 50 uninfected wild females were crossed with 50 wMel (Pud) males.

*OCG, outcross generation.

**TABLE 4:** Induced cytoplasmic incompatibility observed in crosses between uninfected wild *Ae. aegypti* (Pud) females and *w*AlbB-infected *Ae. aegypti* (Pud) males.

Generation	Replicate^#^	No. of eggs laid	No. hatched	Egg hatch rate (%)
OCG*1	1	764	0	0%
	2	955	0	0%
	3	558	0	0%
OCG 2	1	1,168	0	0%
	2	1,363	0	0%
	3	1,033	0	0%
OCG 3	1	768	0	0%
	2	510	0	0%
	3	1,076	0	0%

^#^In each replicate, 50 uninfected wild females were crossed with 50 wAlbB (Pud) males.

*OCG, outcross generation.

### Insecticide resistance

When exposed to the discriminating concentration of DDT (4%) and malathion (0.8%), the percent mortality of both the *Ae. aegypti* (Pud) lines and the wild *Ae. aegypti* females ranged from 0.0 to 5.7% (Table 5). Against deltamethrin 0.03%, the percent mortality of the (Pud) lines and the wild females ranged from 81.4 to 83.6%. The percent mortality of the *Wolbachia*-introgressed *Ae. aegypti* (Pud) lines and of the wild *Ae. aegypti* (Pud) females was almost 100% on exposure to 5x discriminating concentration of malathion and deltamethrin (Table 5). At the diagnostic concentration of temephos (0.02 mg/L), the percent mortality of larvae of both the *Wolbachia*-introgressed *Ae. aegypti* (Pud) lines and the wild *Ae. aegypti* (Pud) line ranged from 61.0 to 70.0%.

**TABLE 5:** Percent mortality of *w*Mel and *w*AlbB *Ae. aegypti* (Pud) lines and wild *Ae. aegypti* (Pud) females on exposure to discriminating/5x concentrations of public health insecticides.

Insecticide and concentration	Strains tested	Treated		Control		Percent mortality
		No. exposed (No. of replicates)	No. dead	No. exposed (No. of replicates)	No. dead	
**Discriminating concentration**						
DDT 4%	*w*Mel (Pud)	300 (12)	6	150 (6)	0	2.0%
	*w*AlbB (Pud)	300 (12)	17	150 (6)	0	5.7%
	Wild (Pud)	300 (12)	11	150 (6)	0	3.7%
Malathion 0.8%	*w*Mel (Pud)	300 (12)	1	150 (6)	0	0.3%
	*w*AlbB (Pud)	300 (12)	0	150 (6)	0	0.0%
	Wild (Pud)	300 (12)	2	150 (6)	0	0.6%
Deltamethrin 0.03%	*w*Mel (Pud)	280 (12)	234	140 (6)	0	83.6%
	*w*AlbB (Pud)	280 (12)	228	140 (6)	0	81.4%
	Wild (Pud)	280 (12)	233	140 (6)	0	83.2%
**5X concentration**						
Malathion 4%	*w*Mel (Pud)	300 (12)	299	150 (6)	0	99.7%
	*w*AlbB (Pud)	300 (12)	300	150 (6)	0	100%
	Wild (Pud)	300 (12)	299	150 (6)	0	99.7%
Deltamethrin 0.15%	*w*Mel (Pud)	300 (12)	300	150 (6)	0	100%
	*w*AlbB (Pud)	300 (12)	300	150 (6)	0	100%
	Wild (Pud)	300 (12)	300	150 (6)	0	100%

## Discussion

ICMR-VCRC in collaboration with WMP has generated two new *Ae. aegypti* (Pud) lines introgressed with the *w*Mel and *w*AlbB *Wolbachia* (Aus) strains for field release and testing. *Wolbachia* strains have many traits that induce a fitness cost and confer fitness benefit on their host mosquito populations. The effects of *Wolbachia* infections on the biological/reproductive fitness characteristics, such as fecundity, egg hatch rate, egg viability, locomotor ability, blood feeding, adult survival, and male mating competitiveness, have been documented (Evans et al., 2009; Turley et al., 2009; Walker et al., 2011; Yeap et al., 2011; Carvalho et al., 2020). Fitness of the *Wolbachia-*introgressed *Ae. aegypti* (Pud) lines are the key factors that would determine their rapid spread into native wild populations when tested in the field. The *Wolbachia*-introgressed (Pud) lines must have at the minimum a similar level of fitness characteristics compared to that of the native wild *Ae. aegypti* populations. This study examined the effects of the *w*Mel and *w*AlbB *Wolbachia* infections on the fitness of the newly generated *Ae. aegypti* (Pud) lines with reference to the wild *Ae. aegypti* (Pud) populations. The maternal transmission efficiency, cytoplasmic incompatibility, and insecticide resistance status of the (Pud) lines were also investigated. It is to be noted that the fitness characteristics that were measured in the laboratory represent correlates of field fitness although they were not the measures of actual fitness, which can be determined only in the field. However, the data generated on the fitness measurements showed that the backcrossed lines are not heavily compromised in terms of these correlates and as such appear suitable to be used as release lines.

The fitness characteristics, such as wing length, fecundity, fertility, and longevity, are highly sensitive to variations in the micro-environment and feeding regimen. Hence, the experiments were carried out with all the five lines under controlled temperature (27 ± 2°C), relative humidity (80%), and rearing conditions (including egg storage, egg hatching, larval rearing, and larval and adult feeding regimens). In terms of physical fitness, that is, wing length, *w*Mel and *w*AlbB infections produced a fitness benefit in the two *Ae. aegypti* (Pud) lines. The wing length of females of both *Wolbachia*-introgressed (Pud) lines was significantly greater than that of the wild (Pud) line. Between the two *Wolbachia*-introgressed (Pud) lines, no significant difference in the wing length was observed. In a previous study, no significant difference in the wing length was observed between *w*AlbB-infected and uninfected *Ae. aegypti* males and females (Axford et al., 2016).

The *w*AlbB (Pud) line had 16% fecundity advantage over the uninfected wild females. In the case of *w*Mel (Pud) line, there was a slight reduction in fecundity (1.06%), but it was not significant compared to that of the wild-type females. The two (Pud) lines showed a comparable egg hatch rate to that of the wild (Pud) line. Joubert et al. (2016) observed that *w*Mel-infected females laid significantly a larger number of eggs than the uninfected females. On the contrary, no significant difference in fecundity was observed between the *w*AlbB-infected and the uninfected *Ae. aegypti* females (Axford et al., 2016; Joubert et al., 2016). The reason for such variations in the results of the studies could be attributed to the different strains of *Wolbachia-*introgressed into *Ae. aegypti* lines with different genomic backgrounds. It has been reported that the same *Wolbachia* strain may have a different effect on the fitness of a strain depending on the host background (Carvalho et al., 2020).

In this study, both sexes of the *w*Mel-infected *Ae. aegypti* (Pud) survived longer than the wild *Ae. Aegypti*, indicating a fitness benefit conferred by the *w*Mel infection. The survival of the *w*AlbB-infected (Pud) females and males was comparable to that of the wild *Ae. aegypti* (Pud) line. As a result, in terms of adult survival, the *w*Mel (Pud) line could be in an advantageous position than the *w*AlbB (Pud) line. In an earlier study, out of the four *Wolbachia* strains tested namely, *w*Mel, *w*AlbA, *w*AlbB, and *w*Au, *w*Mel was the only infection that did not cause a significant reduction in adult female longevity (Ant et al., 2018). *w*Mel- and *w*AlbB-infected *Ae. aegypti* males and females lived longer than the uninfected males and females. When the two infected strains are compared, *w*Mel males and females survived longer than the *w*AlbB-infected males and females (Axford et al., 2016). It has been reported that the survival rate of *Ae. albopictus* females infected with *w*AlbA and superinfected with *w*AlbA and *w*AlbB was higher than that of the uninfected females (Dobson et al., 2002, 2004). There was a 50% reduction in survival rates in *w*MelPoP-infected *Ae. aegypti* line, whereas it was only 10% in the *w*Mel-infected *Ae. aegypti* line, indicating that the *w*Mel infection induced a less fitness cost on adult survival than the life-shortening *w*MelPoP strain (Riegler et al., 2005; Walker et al., 2011). Joubert et al. (2016) reported a higher mean survival time for the *w*Mel- and *w*AlbB-infected *Ae. aegypti* females than the uninfected females, as observed in this study. Increased adult survival is a fitness advantage conferred by an avirulent *w*Mel strain that could facilitate the introgression of the *w*Mel infection into the wild populations upon field releases. In the city-wide field trials, *w*Mel had invaded wild mosquito populations successfully (O’Neill et al., 2018; Indriani et al., 2020; Ryan et al., 2020) and the infection remained stable in the release areas (Frentiu et al., 2014; Hoffmann et al., 2014), whereas the field release of a virulent *w*MelPoP strain in Vietnam failed to invade the wild population due to its life-shortening deleterious effect (Nguyen et al., 2015). In addition to the above factors, male mating competitiveness is one of the important life-history traits that influence the successful establishment of the inherited *Wolbachia* infections into the wild population (Carvalho et al., 2020).

The maternal transmission of *Wolbachia* strain and its ability to induce cytoplasmic incompatibility are the two essential features that must be conserved while considering a line for field release (Fraser et al., 2017). In this study, a near-complete (99–100%) maternal transmission of *w*Mel and *w*AlbB infections was observed in the (Pud) lines. A perfect (100%) cytoplasmic incompatibility was observed in all the crosses between wild-type *Ae. aegypti* (Pud) females and the *w*AlbB or *w*Mel *Ae. aegypti* (Pud) males, except in the first generation of *w*Mel *Ae. aegypti* (Pud) line, where there was an egg hatch rate (viable progeny) of 0.2–1.2%, and on screening, 13 (54.2%) of the emerged adults (*n* = 24) were found positive for *Wolbachia*. This was, however, well below the acceptable level of < 3% (World Mosquito Program [WMP], 2019). As indicated by the results, CI is thus expected to provide a reproductive advantage to the *Wolbachia*-infected females over the uninfected females resulting in the spread of *Wolbachia* among local population (Joshi et al., 2014). In the laboratory experiments carried out elsewhere, *w*Mel-transinfected *Ae. aegypti* displayed perfect cytoplasmic incompatibility and maternal transmission (Riegler et al., 2005; Walker et al., 2011; Joubert et al., 2016). Similar observations were made in *w*AlbB-infected *Ae. albopictu*s (Xi et al., 2005; Axford et al., 2016; Joubert et al., 2016), the secondary vector of dengue and chikungunya viruses. *Aedes albopictus* has been responsible for outbreaks of dengue in India (Tewari et al., 2004; Thenmozhi et al., 2007; Kumari et al., 2011) and also in Madagascar (Ratsitorahina et al., 2008), Hawaii (Effler et al., 2005), Mauritius (Issack et al., 2010), and China (Xu et al., 2007).

Field-released mosquito strains require adequate protection against the insecticides used by the public health program to ensure their survival after the field release. It has been shown that the field releases of susceptible lines into wild populations that are resistant were unable to compete with the wild population and failed to result in *Wolbachia* establishment, whereas field releases of lines that are similar in resistant status to the wild population led to the successful spread of *Wolbachia* infection among the local wild population of *Ae. aegypti* (Garcia et al., 2020). The determination of insecticide resistance profile of the wild-type *Ae. aegypti* (Pud) is also equally important since that will help in deciding where to collect the wild-type mosquitoes for backcrossing experiments to raise the local *Wolbachia*-infected (Pud) lines that will have similar insecticide resistance status as that of wild mosquito population.

In this study, on exposure to discriminating 1X and 5X concentrations, the two *Wolbachia*-introgressed (Pud) lines showed similar level of resistance to deltamethrin and malathion compared to the wild-type *Ae. aegypti*, indicating that the *Wolbachia*-infected *Ae. aegypti* (Pud) lines would tolerate the insecticide pressure in the field after release. The 100% mortality on exposure to 5X concentrations of deltamethrin and malathion indicated a low level of resistance intensity in the two (Pud) lines, as well as in the wild type to the two commonly used insecticides during emergencies in the control program. At the diagnostic concentration of 0.02 mg/L (World Health Organization [WHO], 1981), the resistance level to temephos was similar in both the (Pud) lines and the wild type.

### Summary

The effects of *w*Mel and *w*AlbB infections on *Ae. aegypti* (Pud) lines were assessed in terms of their physical (wing length) and reproductive fitness. The *Wolbachia*-infected *Ae. aegypti* (Pud) lines had greater wing length than the wild uninfected line. With respect to reproductive fitness, *w*AlbB-infected *Ae. aegypti* (Pud) had an advantage of 16% higher fecundity, while *w*Mel *Ae. aegypti* (Pud) showed a comparable fecundity with the uninfected wild females. Both the *w*Mel- and *w*AlbB-infected *Ae. aegypti* (Pud) lines presented a higher egg hatch rate than the uninfected wild (Pud) line. The *w*Mel-infected *Ae. aegypti* (Pud) line lived significantly longer than the *w*AlbB-infected (Pud) line and the uninfected (Pud) wild line. Both the *w*Mel and *w*AlbB strains displayed a complete maternal transmission and induced a strong cytoplasmic incompatibility. The two *Ae. aegypti* (Pud) lines showed a similar level of insecticide resistance compared to the uninfected wild *Ae. aegypti* (Pud) line. The study results showed that the *w*Mel and *w*AlbB infections in the two newly generated *Ae. aegypti* (Pud) lines produced strong cytoplasmic incompatibility, perfect maternal transmission, and favorable biological/reproductive fitness benefits, indicating the suitability of the two (Pud) lines for examination under field release trials. Studies on population replacement and male mating competitiveness have been completed (unpublished results), and the results are in favor of this conclusion.

## Data availability statement

The original contributions presented in this study are included in the article/supplementary material, further inquiries can be directed to the corresponding authors.

## Author contributions

PJ, KG, and SS contributed to conception and design of the study. CS, KG, DP, AS, and VA conducted the experiments. DP and SD performed *Wolbachia* diagnosis. CS and MR supervised the experiment. CS organized the database and wrote the first draft of the manuscript. BV performed the statistical analysis. KG and DP wrote the sections of the manuscript. All authors contributed to manuscript revision, read, and approved the submitted version.
